# Identifying Latent Profiles of Healthy Adults' Biopsychosocial Pain Concepts

**DOI:** 10.1155/prm/5706849

**Published:** 2025-05-09

**Authors:** Catherina Lenhof, Laura Dukek, Linda Wickering, Lena Hitschler, Michael Schneider, Tanja Hechler

**Affiliations:** ^1^Department of Clinical Psychology and Psychotherapy for Childhood and Adolescence, University of Trier, Trier 54286, Germany; ^2^Department of Psychiatry, Psychotherapy and Psychosomatics, Faculty of Medicine, RWTH Aachen University, Aachen 52062, Germany; ^3^Department of Educational Psychology, University of Trier, Trier 54286, Germany; ^4^Department of Psychology, Clinical Psychology and Psychotherapy for Children and Adolescents, University of Münster, Münster 48149, Germany

**Keywords:** biopsychosocial, chronic pain, latent profile analysis, pain concepts, pain science education

## Abstract

**Objectives:** To develop effective, individualized pain science education for people with chronic (primary) pain, underlying pain concepts, defined as the understanding of what pain is, what function it serves, and what processes are thought to underpin it, are essential. Pain concepts and misconcepts of chronic pain can influence its development and maintenance. This study explores whether profiles of healthy adults' biopsychosocial pain concepts can be identified using a newly developed tool, the *biopsychosocial pain concept matrix* (BiPS matrix), and if adults assigned to the profiles differ regarding sociodemographic and pain-related variables.

**Methods:** *N* = 229 healthy adults (75% female, *M* = 22.66 years, SD = 3.61) participated in an online survey. The BiPS matrix assesses biopsychosocial pain concepts through 40 items on the biological, psychological, and social domains combined with the five content dimensions of the common-sense model of self-regulation.

**Results:** A latent profile analysis (LPA) revealed a five-profile solution with distinct patterns of biopsychosocial pain concepts. Participants assigned to Profile 1 demonstrated strongly developed biopsychosocial pain concepts, Profile 2 showed weakly developed concepts, and Profiles 3 to 5 showed different levels of pain concepts. General and neurophysiological pain knowledge differed between profiles, with higher scores being associated with Profile 1 and lower scores with Profile 5. There were no differences in sociodemographic variables in adults assigned to the different profiles.

**Discussion:** Results provide preliminary evidence for distinct profiles of biopsychosocial pain concepts among healthy adults. Further research should replicate these findings in clinical samples to better understand biopsychosocial pain concepts and their use for individualized pain science education.

## 1. Introduction

Chronic pain is widespread and therefore considered a major global health problem with epidemiologic prevalence rates of up to 64.4% [1]. However, definitions of chronic pain vary considerably across studies. While most studies define it as pain persisting for more than 3 months, some apply a threshold of 6 months [1]. Additionally, studies differ in how they define pain frequency, using terms such as “persistent,” “recurrent,” or “at least one pain episode during the last month” [1]. This study adopts a definition based on recommended time durations from previous research, characterizing chronic (primary) pain as occurring at least once per month experienced within the last 6 months or persistent pain [1, 2]. Regardless of these definitional differences, the consequences of chronic pain remain severe as it not only puts a strain on the health system but it also significantly impairs physical and emotional functioning in everyday life: 21% of adults experiencing chronic pain have been diagnosed with depression, 19% have lost their jobs, and 61% were less able or unable to work outside of their homes [3].

The biopsychosocial model provides the foundation for understanding the development and maintenance of chronic pain [4]. This model views chronic pain as a complex interaction of biological (e.g., medication, sleep, surgery), psychological (e.g., cognitions, emotions, behaviors), and social (e.g., healthcare, family, work) variables [5, 6]. As chronic pain arises from the intricate interactions among biological, psychological, and social variables, treatment guidelines recommend an interdisciplinary, multimodal treatment approach [7]. However, despite the ever-growing understanding of chronic pain and its most effective treatment, 40% of patients suffering from chronic pain reported that their pain treatment was insufficient [3] and included a unimodal treatment approach such as prescribing opioid medication with subsequent adverse treatment effects [8–10]. The key question therefore is which factors account for insufficient treatment options for individuals with chronic pain.

One factor that has been extensively discussed is the individuals' and caregivers' pain concept. Moseley and Butler [11] introduced the term pain concepts and differentiated between concepts as “overall mental frameworks” (p. 103), knowledge as “the bits of justified information” (p. 103), and beliefs as “an attitude towards knowledge” (p. 103). In contrast to *pain beliefs* which are referred to as corresponding information specified in a single sentence or statement incorporating what pain means for patients [12, 13], a person's *pain concept* refers to their understanding of what pain is, what function it serves, and what processes contribute to it [14, 15].

Concepts are theory-like knowledge structures that are part of learners' semantic declarative knowledge in long-term memory (see [16] for a review). They can include various knowledge elements, such as facts, assumptions, explanations, or mental images. Due to their relational nature, concepts allow subsuming instances under categories, understanding principles, making predictions, generalizing conclusions, communicating ideas, interpreting and encoding new information during learning, and developing solutions to novel problems [17]. Concepts have been recognized as a central outcome of learning processes, for example, in school, the workplace, and patient education [18]. A review of 40 meta-analyses found that patient education reduced medication use, pain, and visits to medical facilities and significantly improved physiological, physical, and psychological outcomes, as well as patients' general functioning [19]. At least some of these effects can be attributed to the fact that a better conceptual understanding improves patients' self-management, informed decision-making, and treatment adherence even though pain concepts were not assessed in the studies [20, 21].

Concepts are notoriously difficult to measure because learners can acquire different concepts in the same domain independently of each other and at different times [22]. Learners can simultaneously have more and less advanced concepts and even logically incompatible concepts in their long-term memory [23]. Thus, measuring the overall amount of conceptual knowledge (e.g., using a sum score or latent factor) does not suffice. Instead, several subscales are needed to assess which concepts learners have. Furthermore, learners' concepts gradually differ in their strengths and cannot be measured in an all-or-none fashion but require continuous scale scores. Previous empirical studies (e.g., [24, 25]) successfully addressed these measurement problems by measuring several of patients' concepts in a domain such as human memory [24] or rational number concepts [25], each with a continuous subscale. Each learner's conceptual knowledge could then be represented as a profile line of the subscale scores, where each score indicates how strongly a concept is developed in a learner. The studies used latent profile analysis (LPA) to identify latent classes of learners with similar profile lines (i.e., similar combinations of strongly developed or weakly developed concepts). The identification of such classes is an effective data reduction technique and allows for statistical tests of predictors, covariates, or outcomes associated with the class memberships [26].

Given the relational and multidimensional nature of concepts, it is particularly interesting to investigate how biological, psychological, and social concepts of pain are represented in peoples' long-term memory. Focusing on chronic pain patients, various studies have demonstrated that they often adopt a biological concept of their condition, frequently attributing it to tissue damage as the sole biological cause of their pain (e.g., [27]). These misconcepts or biomedical pain concepts contrast evidence showing that physical causes of (chronic) lower back pain are rarely identified [27]. Such misconcepts not only interfere with learning but also possess explanatory power and often compete effectively with scientifically accurate concepts [28, 29]. If patients endorse a biological pain concept, they are more inclined to avoid movement and activity or seek solely medical care [29–31]. The model of misdirected problem-solving suggests that framing chronic pain as a biomedical problem can lead to increased suffering and disability [32]. According to this model, chronic pain patients tend to worry about the causes of their pain and its associated negative consequences, resulting in heightened hypervigilance toward their pain. When chronic pain is framed as a biomedical issue and the patient identifies a solution (e.g., seeking medical care), both pain and worry may temporarily subside. However, if problem-solving attempts fail because no medical cause for the chronic pain is identified, the patient's worry can be amplified. This often leads to a perseverance loop, where the patient cycles from one treatment to another without resolution [32, 33]. But, as outlined previously, the biopsychosocial model shifts the focus from solely examining biological concepts to also considering the patient's psychological as well as social concepts, all of which influence pain experiences and behaviors [5, 6]. Consequently, evaluating, diagnosing, and treating individuals with chronic pain requires a comprehensive approach that integrates psychosocial (e.g., emotions, behaviors) and social factors (e.g., relationships, healthcare) alongside, but not subordinate to, biomedical considerations [34]. Previous research has shown that biological and psychological concepts interact in a way and that chronic pain patients with strong biomedical concepts are less likely to adopt interdisciplinary approaches that foster self-efficacy and encourage personal responsibility for self-management strategies (e.g., developing pain control skills, activity pacing) [35, 36]. Similarly, pain catastrophizing, defined as a cognitive and emotional response to one's pain experience, which leads to the exaggeration of its negative aspects and consequences [34, 37], also negatively influences treatment effectiveness as well as treatment dropout [38]. Pain catastrophizing can include psychological misconcepts, such as “There is nothing I can do to make my pain better,” reflecting a feeling of helplessness and reduced self-efficacy [34, 38]. This illustrates that not only biological, but also psychological pain (mis)concepts can significantly influence treatment outcomes, as they shape patients' engagement with and adherence to therapeutic interventions. The importance of not only recognizing biological concepts but also psychological and psychosocial concepts in chronic pain treatment is also reflected by the finding that chronic pain patients can be differentiated into distinct subgroups: dysfunctional (severe pain, comprised life activities and enjoyment, reduced sense of control, high emotional distress), interpersonally distressed (high degrees of pain and affective distress, low levels of perceived support from significant others), and adaptive copers (low levels of pain, functional limitations, and emotional distress) [35, 39]. Additionally, the subgroup “interpersonally distressed” [35, 39] shows the importance of the social domain of the biopsychosocial model [4]: not only do social activities decrease due to pain, but patients also become increasingly isolated from their social environment as interpersonal relationships are oftentimes focused on the pain [40]. Subsequently, the relationships become strained as friends and families become frustrated and angry. These negative experiences can lead to a perception of a lack of control in pain management [40]. Social pain concepts reflect conceptual knowledge about the interaction between chronic pain, chronic pain development, and social factors such as interactions with friends and families (e.g., “The intensity of pain changes when you argue with your partner,” “Chronic pain can cause you to withdraw from friends, family, or your partner more often”) [41]. Even though it is well established that social factors modulate chronic pain [42], currently available tools focus predominantly on the biological and psychological domain:

Assessment tools, such as the *Neurophysiology of Pain Questionnaire* (NPQ), focus on the biological domain of the biopsychosocial model and examine a person's understanding of the neurophysiological/biological mechanism underlying chronic and acute pain and how and why pain is perceived [43, 44]. The NPQ has been used to identify knowledge gaps in patients, e.g., it could be shown that the inclusion of an “undecided” response option leads to the identification of patients' knowledge gaps, while incorrect responses indicate mistaken beliefs [43].

The *Concept of Pain Inventory* (COPI) [45] is the first tool to assess pain concepts explicitly containing biological aspects *and* psychological aspects of chronic pain [45]. The tool was originally designed for children and has recently been adapted for use in adults, resulting in a 13-item unidimensional questionnaire with each item rated on a 5-point Likert scale from 0 “strongly disagree” to 4 “strongly agree” (e.g., “Item 1: The brain processes lots of details before you feel pain”) [46]. According to the authors, knowledge gaps and existing misconcepts can be identified with the tool to facilitate individualized pain science education [45]. Notwithstanding the advantages of the COPI, it does not contain items regarding social pain concepts. To the best of our knowledge, there is currently no assessment tool that assesses all biological, psychological, and social pain concepts simultaneously.

Pain concepts are shaped not only by biopsychosocial knowledge of pain but also by the illness representation defined as mental models formed by individuals to understand and manage their illness [30]. They are based on lay knowledge, external social environment, and personal experiences, including symptoms and previous coping effectiveness, while also being influenced by personality and cultural background [47]. According to the *common-sense model of self-regulation* (CSM), this illness representation embodies five dimensions: (1) illness identity (label or name as well as the perception of the symptoms, e.g., cancer, flu, etc.), (2) cause (e.g., stress as a cause for heart disease), (3) consequences (experienced or anticipated physical, cognitive, or social disruption), (4) timeline (duration of the illness), and (5) control (e.g., self-versus medical provider) [48]. In a meta-analysis, these dimensions could be shown for 23 illnesses, e.g., multiple sclerosis and acute pain, undermining the stability of the dimensions [30]. Research into chronic pain patients could show that patients could be clustered based on their illness representations assessed with the Revised Illness Perception Questionnaire (IPQ-R) [49] into different subgroups: adaptors with less pain-related consequences and stronger beliefs in personal and treatment control over pain than subgroup two, the nonadaptors [50]. While pain concepts include a person's understanding what pain is, what function it serves as well as its underlying processes [15], illness representations incorporate broader mental models about the overall experience and management of a health condition, with pain as a potential element [30]. Even though the IPQ-R [49] is a promising tool to assess patients' illness representation of chronic pain on the five dimensions, an assessment of patients' biopsychosocial pain concepts has not yet been integrated.

To address this research gap, we developed the *biopsychosocial pain concept matrix* (BiPS matrix) (T. Kloos, unpublished data, April 2019 [29, 41]). The first version of the BiPS matrix was based on an extensive literature review and expert feedback and integrated both the biopsychosocial model and the dimensions according to the CSM [48]. The resulting matrix structure is depicted in Table A1 (translated version without psychometric testing), with the biological, psychological, and social pain concepts being captured at the column level, while the five dimensions are mapped row-wise [29, 41]. An expert survey confirmed the clinical and scientific relevance of the assessment of biopsychosocial pain concepts [41].

An assessment of people's biopsychosocial pain concepts and an identification of resulting biopsychosocial profiles of pain concepts can help identify strongly or weakly developed biological, psychological, and social pain concepts, individual knowledge gaps, and misconcepts which can be targeted subsequently within individualized pain science education. Current research shows that pain concepts are not assessed in studies investigating the effectiveness and mechanisms of pain science education in chronic pain research [51]. By neglecting patients' pain concepts, patients are treated as a homogeneous group without considering qualitative differences in the composition of biopsychosocial pain concepts and thus the individual needs of the patient [26]. Additionally, sociodemographic and pain-related variables can be identified that may predict profile membership [52].

To summarize, no study has thus far investigated profiles of biopsychosocial pain concepts in adults implementing LPA. Therefore, in the current study, we investigated healthy adults' biopsychosocial pain concepts measured with the BiPS matrix. The following research questions were addressed:

  (1) Are there latent classes of healthy adults differing systematically in their profiles of biopsychosocial pain concepts?
  (2a) Do people assigned to latent classes differ in regard to sociodemographic variables such as sex and age?
  (2b) Do people assigned to latent classes differ in regard to pain-related variables, such as pain intensity?
  (2c) Do people assigned to latent classes differ in their self-rated general pain knowledge and neurophysiological pain knowledge?

## 2. Materials and Methods

### 2.1. Participants and Study Procedures

In total, *N* = 229 (75% female) adults aged between 18 and 50 years (*M = *22.66 years, SD = 3.61) were included in the analysis. The data used in this study were collected online as part of a research project between April and May 2021 using the survey software *Unipark* of the company *Questback* (L. Hitschler, unpublished data, August 2021). Participants gave written informed consent of participation after having been informed about the use of data, the aim of the study, anonymity, and the possibility of stopping the study at any point. The present study was approved by the Ethics Committee of the University of Trier (no. 04-2021) (T. Hechler, Prof. Dr. and C. Lautwein, unpublished document, April 2021).

### 2.2. Measures

#### 2.2.1. BiPS Matrix

We developed the BiPS matrix to assess patients' pain concepts based on both the biopsychosocial model [4] and the dimensions according to the CSM [48]. This results in a matrix structure (see Table A1), with the biological, psychological, and social pain concepts being captured at the column level, while the five content dimensions are mapped row-wise. The BiPS currently exists in two versions (for children [29] and adults [41]).

The original version of the BiPS matrix contained 72 items (T. Kloos, unpublished data, April 2019) and was tested in a small recent validation study with *N* = 47 adults (*M* = 33.55 years, range 17–68 years, 70.13% female). An excellent internal consistency of the BiPS matrix of *α* = 0.95 was revealed. The internal consistencies for the vertical domains (biopsychosocial) were acceptable to excellent (biological scale: *α* = 0.73, psychological scale: *α* = 0.92, social scale: *α* = 0.88). For the horizontal dimensions as defined in the CSM [48], the internal consistencies with a range of 0.72 < *α* < 0.90 were also in an acceptable to excellent range (cause: *α* = 0.80, consequences: *α* = 0.72, type of disorder: *α* = 0.75, timeline: *α* = 0.78, control: *α* = 0.90). We found significant correlations between the total score of the BiPS matrix and the self-assessed previous knowledge (*r* = 0.33, *p* < 0.01). Subjects without chronic pain (69.2%) showed a higher total score in the BiPS matrix than those with chronic pain (*t* (45) = 3.34, *p* < 0.01, *d* = 1.01) (T. Kloos, unpublished data, April 2019). The validation study also compared the items of the BiPS matrix to those of already existing, validated questionnaires (e.g., the NPQ-d [44]), and adapted the BiPS matrix items accordingly. In a successive process, we reduced the questionnaire to 63 items. Afterward, we presented the tool to interdisciplinary experts who rated items on relevance and usefulness [41]. The experts attested to the high relevance and usefulness of the BiPS matrix, and experts' comments were used to further reduce and adapt the items for adults and for use in children and adolescents children [29]. The present version for adults, which was used for this study, consists of 40 items (see Table A1). The BiPS matrix uses a scale ranging from 0 to four points, with the “neither/nor” category being assigned more points than an incorrect answer. The choice of the “neither/nor” answer indicates more adequate knowledge compared to an incorrect answer, which reflects inadequate knowledge. For correct evaluation of the items, four points are assigned for strong agreement, three points for agreement, two points for “neither/nor,” one point for disagreement, and 0 points for strong disagreement. For the 12 reversed items, this coding is applied in the opposite direction. In total, it is possible to achieve an overall score between 0 and 160 points, with a higher score indicating a more developed scientific concept of pain.

#### 2.2.2. NPQ-d

The German version [44] of the NPQ, the NPQ-d, was investigated in *N* = 169 chronic pain patients (59.8% female, *M* = 51.52 years) and *N* = 122 physiotherapists (41.8% female, *M* = 37.31 years), showing a moderate to low internal consistency of *α* = 0.52 in the patients' sample and *α* = 0.62 in the physiotherapists' sample. Regarding construct validity, physiotherapists scored significantly higher than their patients (*p* < 0.01). Additionally, test–retest reliability was high (ICC = 0.88; 95% CI = 0.82–0.93).

We used the NPQ-d for our study to assess the neurophysiological knowledge of the participants calculating the mean value of the NPQ-d responses. We also included it in post hoc analyses in regard to research question 2c to validate the profiles and describe them more in detail.

#### 2.2.3. Sociodemographic and Pain-Related Questionnaire

##### 2.2.3.1. Demographics

The sociodemographic questionnaire, specifically developed for this study, assessed demographic data like age, sex, university major, and housing situation (see Appendix A).

##### 2.2.3.2. Self-Rated General Pain Knowledge

We measured self-rated general pain knowledge on a visual analogue scale (“On a scale from 0 (no general pain knowledge) to 100 (very high general pain knowledge), how high would you rate your general pain knowledge?”).

##### 2.2.3.3. Pain-Related Variables

The current study focused on the following pain characteristics: participants rated their previous pain education with the following item: “Do you have professional experience in the field of pain or have you attended a lecture or seminar on the subject of pain?” The respond options included “no,” “yes, professional experience,” and “yes, attended a lecture/seminar.” We also asked participants to report whether friends or family members were suffering from chronic pain (“Does anyone in your family, circle of friends, or immediate environment suffer from recurring or chronic pain (i.e., pain at least once a month in the last 6 months or persistent pain)?”). The response options were “no,” “I don't know,” “yes, but I don't notice much of it,” and “yes and I am dealing with the topic.” To assess participants' acute or chronic pain status, they reported if they were suffering from acute pain (“yes”/“no” question). If so, they rated their pain intensity (NRS: 1 = “weakest pain,” 10 = “most severe pain”) and where it was located (“Where is your current pain localized (multiple answers possible)?”; response options: “head,” “abdomen,” “back,” “joints,” “extremities,” and “other” with an option for free text. We then asked participants if they suffered from chronic pain, defined as pain at least once a month in the last 6 months or persistent pain [1, 2]. If a participant responded with “yes,” further questions on the quality of the chronic pain appeared. First, analogous to the questions related to acute pain, chronic pain intensity in the last four weeks was measured (NRS: 1 = “weakest pain,” 10 = “most severe pain”) and where it was located. Second, participants rated the frequency of experienced chronic pain: “How often did your (main) pain generally occur (on average) in the last 4 weeks?” A definition of “main pain” was provided (If pain occurs in several places, the main pain is the pain that significantly affects you.) and the response option included “no pain in the last 4 weeks,” “pain less than once a day,” “pain maximum once a day,” “pain is always present except for rare pain-free moments and pain-free sleep,” and “pain is constantly present” (see Appendix A).

### 2.3. Statistical Analysis

#### 2.3.1. Data Preparation

A total of *N* = 242 adults participated in the study, of which *n* = 5 had to be excluded due to incomplete participation or ineligibility. We checked the time needed to complete the questionnaire for outliers to detect invalid data. In particular, we excluded participants who took less than five (*n* = 5) or more than 25 min (*n* = 2) from further analyses to ensure data quality and comparability by removing responses that were likely rushed and careless (< 5 min) or distracted and inconsistent (> 25 min). In addition, one participant had to be excluded for providing the same response on all questionnaire items. Our dataset had no other missing values due to the online survey software used for the data collection. Therefore, we included a total of *N* = 229 people in the subsequent analyses.

We recoded inverted items, so participants received four points for correctly rejecting the item. Items of the NPQ-d [44] were coded to 0 for every incorrect or undecided answer and 1 for every correct answer. We calculated descriptive statistics of demographic and pain-related variables as well as the overall and subscale scores for the BiPS matrix.

#### 2.3.2. LPA

Firstly, regarding *research question 1—Are there latent classes of healthy adults differing systematically in their biopsychosocial profiles of pain concepts?* we performed an LPA following the guidelines proposed by Spurk and colleagues [52]. We specified the three BiPS matrix subscale scores for biological, psychological, or social pain concepts (i.e., knowledge on biological, psychological, and social processes) as indicators of a latent class variable. The LPA treats class membership as an unobserved categorical variable, and the class membership probabilities are estimated model parameters. More specifically, a person-centered approach groups individuals based on shared characteristics that differentiate the members of different profiles, while a variable-centered approach (e.g., factor analysis) groups similar variables and items [53].

The indicators were standardized before the analyses to aid model estimation. The random seed was set to 1234 to ensure replicability. The LPA model was estimated using the package tidyLPA [54] as an interface to mclust for Gaussian mixture modeling [55] in the statistic software R. SPSS (version 27) was used for the subsequent analyses of class differences in covariates. In our case, the classes were characterized by their level of knowledge regarding different indicators [56, 57]. A series of LPA models with increasing numbers of classes was estimated, starting with a one-class model. The number of classes was increased until problems in convergence or under-identification of the model occurred [56].

The decision for the number of classes in the final model was based on information criteria and significance tests. The three most common information criteria for determining model goodness of fit used in this statistical analysis were the Bayesian information criterion (BIC), the adjusted BIC (aBIC or SABIC), and the Akaike information criterion (AIC) [52, 56, 58]. In contrast to other methods for testing model goodness of fit, these three indices penalize for the number of model parameters to ensure the estimation of parsimonious models and avoid overfitting [58]. The model with the lowest indicator values in all parameters (in BIC, aBIC/SABIC, and AIC) fits the empirical data best.

In addition to considering the information criteria, the *Vuong–Lo–Mendell–Rubin likelihood ratio test* (LMRT) and *parametric bootstrapped likelihood ratio test* (BLRT) [52, 58, 59] were used to determine whether a model fit can be improved by increasing the number of latent classes [56]. Both tests compare whether models with *k* and *k* + 1 latent classes differ in their model fit. If this is not the case, the more parsimonious model with fewer latent classes is preferable [60].

The quality of the estimated model was also evaluated based on the entropy, which indicates the classification accuracy as a value between zero and one, with higher values indicating a better accuracy [61].

#### 2.3.3. Indicator Differences Within and Between Classes

We exported the persons' indicator scores and model estimated class probabilities to SPSS for further examination. The number of persons in each class was calculated based on each person's most likely class membership. We then inspected the classes' indicator mean scores to aid the interpretation of the classes and their differences. For each latent class, *repeated-measures analysis of variance* (ANOVA) were conducted to test whether the mean scores of the three indicators (biological, psychological, and social) differed significantly *within* that class. This approach allowed us to assess the balance of the three types of concepts in each latent class. Furthermore, an *analysis of means* (ANOM) was calculated separately for each class and indicator to test whether the class members' indicator mean differed from the sample mean on that indicator, thus demonstrating that the respective concepts were stronger or weaker for that class than the overall sample. To compare whether the three indicator variables (biological, psychological, and social) differ significantly *across* the five classes, three separate one-way ANOVAs were conducted. In each ANOVA, one of the indicator variables served as the dependent variable (biological, psychological, or social), and the class membership (five levels, one for each class) was the independent variable. Bonferroni post hoc tests were subsequently applied to identify specific pairwise differences between the classes for each indicator variable. Since three separate ANOVAs were performed, a Bonferroni correction was applied to adjust the alpha-level and control for the increased risk of Type I error.

##### 2.3.3.1. Class Differences in Sociodemographic Characteristics

To investigate *research question 2a—Do people assigned to latent classes differ in regard to sociodemographic variables such as sex and age?*—classes were characterized by extracting the descriptive characteristics of the variables age and sex. Subsequently, a chi-square test for the dichotomous variable sex was performed, and a Kruskal–Wallis test for the non-normally distributed variable age. Furthermore, a multiple linear regression model was used to examine the relation between the demographic variables, age and sex (predictors), and the probability of class membership (criterium) for every profile.

#### 2.3.4. Class Differences in Pain Knowledge and Pain-Related Variables

Regarding *research question 2b—Do people assigned to latent classes differ in regard to pain-related variables*?—the same procedure was applied, focusing on pain-related variables: Chi-square tests for the dichotomous variables (acute/chronic pain status, previous pain education) and a Kruskal–Wallis test for the ordinal-scaled variable “pain in the social environment” were used. Additionally, one-way ANOVAs were performed to investigate whether the classes differ in interval-scaled pain-related variables, including frequency of chronic pain, intensity of chronic pain, and intensity of acute pain. For each ANOVA, the dependent variable was one of the pain-related measures, while the independent variable was profile membership (five levels). Bonferroni post hoc tests were applied to identify specific pairwise differences between the classes. As with the previous analyses, the alpha-level was adjusted to control for multiple comparisons. Separately for each latent class, a multiple linear regression model was used to examine whether the pain-related variables predicted the persons' probabilities of being in that class. Focusing research question 2c—*Do people assigned to latent classes differ in their self-rated general pain knowledge and neurophysiological pain knowledge?* classes were compared regarding their self-estimated previous knowledge and their neurophysiological knowledge using two separate ANOVAs and Bonferroni post hoc tests, followed by multiple linear regression with self-estimated previous knowledge and neurophysiological knowledge (NPQ-d mean value) as predictors and probability of class membership of each class as criterium.

## 3. Results

Descriptive statistics of demographic and pain-related variables were calculated, as well as means for each subscale of the BiPS matrix (see Table 1).

### 3.1. Research Question 1—Are There Latent Classes of Healthy Adults Differing Systematically in Their Profiles of Biopsychosocial Pain Concepts?

**Figure 1:**
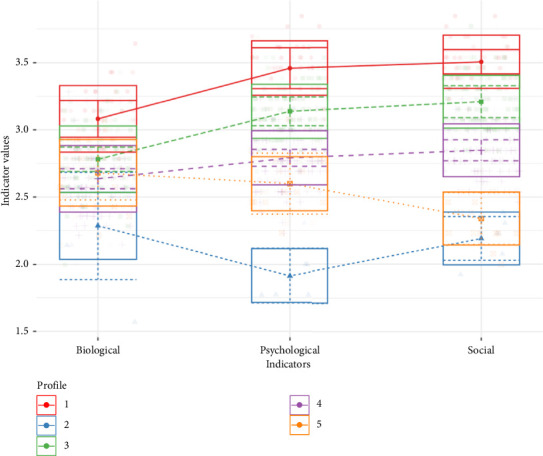
Box plots of the indicator means and standard deviations for the five latent classes.

The model estimation started with the null model, which only has one class, and increased stepwise with *k* + 1 classes. The estimation was stopped after evaluating models with up to nine classes, as the model fit deteriorated gradually with adding six or more classes without improving compared to the previously estimated profiles. For every number of estimated classes, two different models were considered: Model 1 with equal variances across the classes and fixed covariances to zero, and Model 6 with varying variances as well as covariances were estimated. Fit statistics (Table 2) supported the five-class solution based on AIC, BIC, SABIC, BLRT, and LMR. The six-class model did not outperform the five-class model according to the LMRT (*p* = 0.9), whereas the five-class solution fits better than the four-class solution (*p* = 0.01). Based on these findings, we concluded that a five-class solution provided the best fit (Figure 1). Even though the smallest class (Profile 2) only consisted of *n* = 6 healthy adults, the authors decided on the five-class solution based on the statistical indicators as well as the finding that particularly Profile 2 remained consistently small (*N* = 17) even when a four-class solution was considered. Furthermore, Profile 2 showed a very distinct biopsychosocial pain concept compared to the other profiles, which was worth exploring more in detail.

The five-class solution had an entropy of 0.78, indicating an acceptable precision for assigning individuals to their appropriate class, even though the recommended lower boundary of 0.80 was not reached [52].

#### 3.1.1. Characterization of the Class Mean Profiles

**Figure 2:**
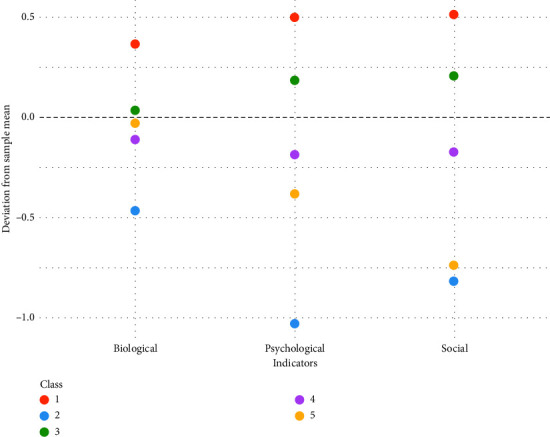
Deviation of the class mean from the sample mean by indicator variable and latent class.

Each participant was assigned to one of the five classes according to their highest probability of class membership. The first profile consisted of 13.97% of the sample (*n* = 32) and showed higher values on the biological, psychological, and social variables than other profiles (see red line in Figure 1 and Table 3). The repeated-measures ANOVA showed that within this profile, the psychological and the social pain indicators were significantly higher than the biological indicator (*F* = 40.89, *p* < 0.01). The plot of the class mean profiles (ANOM; see Figure 2), which illustrates the deviation of means of each profile from the overall mean in each indicator variable, showed that people being assigned to Profile 1 had significantly high values for all three pain concepts (*p* < 0.01). Thus, people in this class showed a mean biological pain concept of *M* = 3.11 (SD = 0.23), which is significantly higher than the mean over all participants (*M* = 2.74, SD = 0.30). The same was shown for the psychological pain concept with *M* = 3.47 (SD = 0.20) versus the overall psychological mean *M* = 2.97 (SD = 0.37) as well the social pain concept with *M* = 3.53 (SD = 0.16) compared to the overall mean *M* = 3.01 (SD = 0.38). We label this profile *strongly developed biopsychosocial pain concept*.

The second latent class comprised 2.62% of the sample (*n* = 6) and was characterized by low scores in all three indicator variables (see blue line in Figure 1 and Table 3). The repeated-measures ANOVA showed no differences between the biological, psychological, and social concepts within this class (*p* > 0.5). The plot of the class mean profiles (see Figure 2), however, showed that the mean pain concepts were significantly lower as compared to the overall means with *M* = 2.27 (SD = 0.21) for the biological, *M* = 1.94 (SD = 0.21) for the psychological, and *M* = 2.19 (SD = 0.17) for the social concept with *p* < 0.01 for all the subscales. Therefore, we call this profile *weakly developed biopsychosocial pain concept*.

The third class consisted of 33.19% of the sample (*n* = 76) and showed moderate values in all three indicator variables (biological, psychological, social) (see green line in Figure 1 and Table 3). Within this class, significant differences between the three indicators were found with the biological being lower than the psychological and social concept (*F* = 89.59, *p* < 0.01). The plot of the class mean profiles (see Figure 2) showed that the psychological and social (*pp* < 0.01) indicators were significantly higher than the sample overall means. We refer to the third profile as *psychosocially dominated pain concept*.

The fourth class consisted of 44.5% of the sample (*n* = 102). Like the third class, it was characterized by moderate values in the biological, psychological, and social pain concepts (see purple line in Figure 1 and Table 3), whereas within the class the biological concept appeared to be significantly lower than the other concepts (*F* = 26.65, *p* < 0.01). The plot of the class mean profiles (see Figure 2) revealed that all indicator means were significantly below the overall sample means (*p* < 0.01). Therefore, we label this profile as *moderate biopsychosocial pain concept*.

The fifth class consisted of 5.68% of the sample (*n* = 13) and showed an overall weakly developed biopsychosocial pain concept (see orange line in Figure 1 and Table 3). Within this class, the mean of the social indicator was significantly lower than the biological and psychological mean (*F* = 12.72, *p* < 0.01). The plot of the class mean profiles (see Figure 2) showed that both the psychological and social concepts were significantly below the sample average (*p* < 0.01). We call this profile *weakly developed psychosocial pain concept*.

Comparing the classes regarding the indicator variables using a one-way ANOVA revealed a significant main effect (*F* = 29.56, *p* < 0.01, partial *η*^2^ = 0.345) in the biological indicator. A Bonferroni post hoc test was carried out to investigate the specific group differences: It showed significant differences between all the classes (*p* < 0.01), except Profile 3 *psychosocially dominated pain concept* and Profile 5 *weakly developed psychosocial pain concept* (*p*=1.0), as well as Profile 4 *moderate biopsychosocial pain concept* and Profile 5 *weakly developed psychosocial pain concept* (*p*=1.0). Thus, the values in the biological indicator between these classes are more homogenous than between the other classes. With regard to the psychological indicator, a more heterogeneous result was revealed: All five classes differed significantly from each other in their psychological pain concept (*F* = 151.49, *p* < 0.01, partial *η*^2^ = 0.730) with Profile 1 *strongly developed biopsychosocial pain concept* having generally higher and Profile 2 *weakly developed biopsychosocial pain concept* having lower scores in the psychological indicator than other classes. In the social indicator, the ANOVA showed a significant main effect (*F* = 196.36, *p* < 0.01, partial *η*^2^ = 0.778). The Bonferroni post hoc showed that all classes differed significantly from each other in their values of the social indicator, except Profile 2 *weakly developed biopsychosocial pain concept* and Profile 5 *weakly developed psychosocial pain concept* (*p*=1.0). The social concept in these two classes was equally low.

### 3.2. Research Question 2a—Do People Assigned to Latent Classes Differ in Regards to Sociodemographic Variables?

The five latent classes' sociodemographic information can be found in Table 3. Males and females were equally distributed across the five classes (*χ*^2^ = 4.15; *p*=0.84), and participants in the five classes did not differ in age (*H* = 2.55, *p*=0.64). Gender and age did not predict the probability of class membership for any of the classes (see Table 4).

### 3.3. Research Question 2b—Do People Assigned to Latent Classes Differ in Regard to Pain-Related Variables?

One-way ANOVAs were used to test whether people assigned to the latent classes differed in the pain-related variables (frequency of chronic pain, intensity of acute/chronic pain). The results indicated tendencies but no statistically significant differences between the classes (*F* = 1.59, *p*=0.09). Members of the five classes did also not differ in terms of acute pain status (*χ*^2^ = 15.31; *p*=0.05), chronic pain status (*χ*^2^ = 3.66, *p*=0.45), previous pain education (*χ*^2^ = 10.3, *p*=0.25), or pain in the social environment (*H* = 5.35, *p*=0.25). The pain-related variables did not predict the probability of class membership significantly (see Table 3). For the probability of class membership in Profile 1 *strongly developed biopsychosocial pain concept*, the variable *frequency of chronic pain* showed a marginally nonsignificant result (*p*=0.02) when adjusting the alpha-level to *p*=0.016 to avoid alpha-error accumulation. This indicates a tendency that a higher frequency of chronic pain was related to a higher probability of being a member of Profile 1 *strongly developed biopsychosocial pain concept* (see Table 3).

### 3.4. Research Question 2c: Analysis of Pain-Related Knowledge Variables

Members of the five classes differed significantly in their neurophysiological pain knowledge assessed with the NPQ-d (*F* = 3.89, *p* < 0.01, partial *η*^2^ = 0.065) (see Table 2). Bonferroni post hoc tests revealed that differences in the neurophysiological knowledge occurred between participants with Profile 1 *strongly developed biopsychosocial pain concept* and Profile 5 *underdeveloped psychosocial pain concept* (*p* < 0.01) and participants with Profile 3 *psychosocially dominated pain concept* and Profile 5 *weakly developed psychosocial pain concept* (*p*=0.04). Specifically, participants with Profile 1 *strongly developed biopsychosocial pain concept* and Profile 3 *psychosocially dominated pain concept* displayed higher neurophysiological pain knowledge than participants with Profile 5 *weakly developed psychosocial pain concept* (see Table 2). The multiple regression model revealed that higher values in neurophysiological knowledge were related to a higher probability of being a member in Class 1 *strongly developed biopsychosocial pain concept* and to a reduced probability of being a member in Class 5 *weakly developed psychosocial pain concept* (see Table 3).

The ANOVA using self-rated general pain knowledge as dependent variables also revealed significant results (*F* = 2.92, *p* < 0.05, partial *η*^2^ = 0.050). Bonferroni post hoc tests showed that Profile 1 *strongly developed biopsychosocial pain concept* and Profile 4 *moderate biopsychosocial pain concept* differed significantly with Profile 1 *strongly developed biopsychosocial pain concept* having the highest self-rated general pain knowledge compared to all other classes (see Table 2). In line with that, higher values of self-rated general pain knowledge were related to an increased probability of being a member in Class 1 *strongly developed biopsychosocial pain concept* (see Table 3), but the variable did not predict the probability of profile membership for the other four profiles.

## 4. Discussion

### 4.1. Summary of Key Findings

The present study aimed to identify profiles of biopsychosocial pain concepts in healthy adults via LPA using the biological, psychological, and social subscale of the BiPS matrix [41]. The identified five latent classes were characterized based on their relationships with demographic and pain-related variables. Furthermore, the profiles were analyzed in regard to their association with other measures of pain-related knowledge such as the NPQ-d [44] and self-rated general pain knowledge. A five-profile solution was found to represent the data best. The conceptual understanding of pain in Class 1 *strongly developed biopsychosocial pain concept* was characterized by high, above average, scores across biological, psychological, and social domains. Class 2 weakly developed biopsychosocial pain concept showed pain knowledge well below the general average across all three domains, with psychological and social knowledge in particular being poorly developed. Class 3 psychosocially dominated pain concept showed a biopsychosocial pain knowledge above average, with psychosocial pain knowledge being more distinct than the biological knowledge. Class 4 moderate biopsychosocial pain concept was characterized by lower biological compared to psychosocial pain-related knowledge, with all of them slightly below average. Class 5 weakly developed psychosocial pain concept showed a biologically dominated knowledge of pain, within the general average, whereby both the psychological and social pain knowledge appeared to be significantly below the overall average.

No differences between the profiles could be found in sociodemographic variables (research question 2a); however, pain-related variables were marginally associated with class membership (research question 2b). Adults assigned to Class 1 *strongly developed biopsychosocial pain concept* showed a high frequency of chronic pain and adults assigned to Class 2 weakly developed biopsychosocial pain concept a low frequency of chronic pain compared to other profiles. Class 3 psychosocially dominated pain concept was characterized by the highest intensity of chronic pain. Class 4 moderate biopsychosocial pain concept showed no significant associations with pain-related variables. In Class 5 weakly developed psychosocial pain concept, members showed a higher prevalence of chronic pain than acute pain. Better self-rated general pain knowledge and neurophysiological knowledge (research question 2c) were associated with an increased probability of being a member in Class 1 *strongly developed biopsychosocial pain concept.* Better neurophysiological knowledge reduced the probability of being a member in Class 5 weakly developed psychosocial pain concept.

### 4.2. Discussion of the Five Identified Profiles

This LPA study is the first to identify profiles of biopsychosocial pain concepts in healthy adults using the BiPS matrix [41], while also examining the role of various covariates contributing to profile membership.

Five latent profiles that differ in the conceptual pain knowledge were found. While biopsychosocial pain knowledge independently of the domain was significantly more developed in Profile 1 *strongly developed biopsychosocial pain concept* and Profile 3 *psychosocially dominated pain concept*, the biological domain appeared to be the least distinctive as it showed less pronounced variability compared to the psychological and social domains which are more prominent, e.g., in Profiles 3 *psychosocially dominated* and 4 *moderate biopsychosocial pain concept*. Profile 2 *weakly developed biopsychosocial pain concept* was characterized by an overall weaker conceptual pain knowledge with the psychological and social knowledge in particular being poorly developed. Profile 4 *moderate biopsychosocial pain concept* showed almost equally distributed pain knowledge for the three domains slightly below the overall average. Profile 5 *weakly developed psychosocial pain concept* represented a biological dominated conceptual pain knowledge within the general average, whereby both the psychological and social pain knowledge appeared to be significantly below the overall average.

The identification of five different profiles (with different sample sizes ranging from *n* = 6 in Profile 2 to *n* = 102 in Profile 4) within such a homogeneous sample of young (female) adults is intriguing and suggests that the profiles may be more pronounced once heterogeneous samples will be assessed. It also suggests that conceptual knowledge of pain varies profoundly even in such a homogeneous sample. The finding that most adults were assigned to Profile 3 *psychosocially dominated pain concept* may be explained by the fact that the majority were psychology students and hence well trained in knowledge of psychological and social factors.

Previous research has also highlighted the clinical value of LPA for enhancing the understanding of clinical phenotypes, illness representations, and risk factors associated with pain localization, as well as psychosocial factors, influenced through treatment [62–65]. Based on the Brief IPQ (B-IPQ) [49, 66], three distinct classes of patients with chronic neck pain were identified: mildly, moderately, and severely affected. The severely affected subgroup exhibited the highest scores for disease consequences and duration/timeline, alongside the lowest scores for perceived control [67]. The authors concluded that identifying latent classes based on the B-IPQ [49, 66] could enhance clinical decision-making and individualized treatment: their study suggests that patients' in the mildly affected group may best profit from a single session of advice, moderately affected patients might benefit from formal physiotherapy, while the severely affected group could require targeted multimodal therapies [67]. Focusing on our study, identifying latent profiles of biopsychosocial pain concepts using the BiPS matrix could enhance treatment decisions in a similar fashion as with the B-IPQ: interventions such as pain science education could be tailored to the patient's individual profile which reflects in detail the level of conceptual knowledge within each domain.

If future studies will replicate the five profiles in more heterogeneous samples, this will illustrate the variance in peoples' knowledge structures and will allow us to identify differences in adults' biological, psychological, and social pain concepts. This information could then be transferred to the conceptualization of individualized pain science education.

### 4.3. Discussion of the Association Between the Identified Profiles and Pain-Related and Sociodemographic Variables

Adults assigned to the five classes did not differ in sociodemographic variables, aligning with previous findings for the NPQ that also revealed no differences in sex and age [68]. Even though ANOVAs did also not show significant differences between the classes in terms of frequency, intensity, and prevalence of chronic and acute pain, the comparison of descriptive scores indicated a tendency: Participants with Profile 1 *strongly developed biopsychosocial pain concept* and Profile 3 *psychosocially dominated pain concept*, characterized by strongly developed pain concepts, exhibited higher frequencies of chronic pain and reported the highest levels of chronic pain intensity. This finding is in line with previous research on pain knowledge in cancer patients revealed that patients experiencing pain had significantly greater knowledge about pain and its management compared to pain-free patients [69]. Patients reporting higher pain intensity scores also displayed greater understanding of pain and its management. It remains uncertain whether increased perceived pain frequency or intensity drives a deeper engagement with the mechanisms of pain development and maintenance, potentially fostering a stronger biopsychosocial understanding. In the present study, we did not assess treatment status. In fact, the high rates of self-reported chronic pain (41.5%) in this sample were unexpected, although it aligns with other research types on students revealing chronic pain prevalences in at least one location reaching from 30.2% up to 64.4%—especially in female students [1, 70–72]. Future research should assess students' chronic pain as well as treatment status to better understand vulnerability for chronic pain in this sample along with their knowledge structures.

### 4.4. Discussion of the Association Between the Identified Profiles and Pain Knowledge Variables

Differences in the neurophysiological knowledge assessed with the NPQ-d [44] were found across the five profiles. Neurophysiological knowledge was associated with the biological domain in each profile which led to significant differences between Profile 1 *strongly developed biopsychosocial pain concept,* Profile 5 *weakly developed psychosocial pain concept*, and Profile 3 *psychosocially dominated pain concept*. Associations between the NPQ-d [44] with the psychological and social indicator could not be shown.

Notably, the mean values of the NPQ-d [44] across the profiles do not align completely with the observed mean values of the biological domain in the BiPS matrix. For instance, Class 5 weakly developed psychosocial pain concept shows the lowest average NPQ-d [44] score but does not simultaneously exhibit the lowest mean value in the biological BiPS matrix domain. This discrepancy suggests that the biological scale of the BiPS matrix [41] extends beyond neurophysiological and nociceptive components, potentially incorporating additional dimensions such as the function of chronic pain.

Profile 1 *strongly developed biopsychosocial pain concept* and Profile 3 *psychosocially dominated pain concept*, however, demonstrate high scores on both the NPQ-d [44] and the biological domain, as well as on the psychological and social domains. Previous research on pain education, utilizing the NPQ alongside other questionnaires, has indicated that education contributes to an increase in biopsychosocial knowledge [73]. This aligns closely with the conceptual pain knowledge patterns observed in Profile 1 *strongly developed biopsychosocial pain concept* (13.9%) and Profile 3 *psychosocially dominated pain concept* (33.19%), representing almost half of the present sample. The study population, primarily composed of psychology students, may be in parts well-educated on pain-related topics, potentially explaining the high scores observed in both the biopsychosocial and NPQ-d [44] domains.

A notable strength of the present study is the use of the BiPS matrix, marking the first integration of pain-related knowledge across biological, psychological, and social domains. This approach offers a more nuanced understanding of people's knowledge structures.

### 4.5. Limitations

While this is the first study to identify latent profiles of adults' pain concepts, our results should be interpreted in the context of the following limitations: Firstly, due to the relatively small sample size (*N* = 229), conclusions based on the present data must be considered carefully. Spurk et al. [52] identified a sample size of 500 for an LPA as reasonable leading to enough accuracy in identifying the correct number of profiles. Future studies should therefore aim to include a larger sample. Secondly, the present sample was selective with respect to age and education: We investigated a well-educated, predominantly female young adult sample. This limits the ability to analyze sociodemographic differences, highlighting the need for an investigation of a more heterogeneous sample. Given that we found five profiles in our homogeneous sample, we expect to see more pronounced profiles when heterogeneous samples are investigated. In addition, clinical samples suffering from (diagnosed) chronic (primary) pain are needed to enhance understanding of their biopsychosocial pain concepts. Even though an unexpectedly large proportion of participants in the present study reported chronic pain (41.5%), the present findings cannot be transferred to the clinical context. Third, when our findings are replicated in future studies, we recommend an assessment of a broader range of pain-related variables, including social and psychological factors (e.g., emotional distress or the social consequences of pain), to improve the prediction of class membership. Based on prior research, it is also advisable to account for variables including headache occurrences, emotional and behavioral difficulties, comorbidities, pain catastrophizing, physical health, and activity levels [62, 64, 65]. The CSM content dimensions [30] integrated into the BiPS matrix [41] could also be taken into account or even a combination of both models (biopsychosocial and CSM [30]). This would result in an even more differentiated consideration of adults' pain concepts but was beyond the scope of this study.

### 4.6. Conclusion and Future Directions in Research and Clinical Practice

We were able to identify five profiles in adults' pain concepts based on the BiPS matrix [41]. Participants in the five latent classes differed particularly regarding their pain-related neurophysiological knowledge and self-rated general pain knowledge, while pain-related variables were not associated with the profiles. Future research should explore whether these profiles can be replicated in more heterogeneous and clinical samples and whether predictor and covariates such as pain-related variables (e.g., chronic/acute pain status, previous pain education, and pain in the social environment) can be identified. Additionally, it would be valuable to investigate whether individuals with different profiles, particularly those with weakly developed profiles, could benefit from individualized pain science education and whether changes in conceptual knowledge can be effectively captured using the BiPS matrix [41]. Pain concepts have thus far not been investigated as an outcome when determining the efficacy of pain science education [51]. These questions offer promising directions for advancing our understanding of biopsychosocial pain concepts and pain science education.

## Figures and Tables

**Table 1:** Descriptive statistics and frequencies of the sample (*N* = 229).

	*M*	SD	Min	Max	*n*	%
BiPS total score (0–160)	112.76	11.19	76	140	229	100
Indicator variables						
Biological subscale (0–4)	2.74	0.298	1.57	3.64	229	100
Psychological subscale (0–4)	3.85	0.367	1.77	3.85	229	100
Social subscale (0–4)	3.01	0.382	1.92	3.85	229	100
Demographic variables						
Age	22.66	3.61	18	50	229	100
Sex					229	100
Female					172	75.11
Male					56	24.45
Diverse					1	0.44
Study subject					229	100
Psychology					179	78.17
Law					1	0.44
Business administration					2	0.87
German studies					2	0.87
Educational science					9	3.93
Physiotherapy					12	5.24
Others					24	10.48
Pain characteristics						
Previous pain education					229	100
No					143	62.45
Yes (professional experience)					30	13.10
Yes (seminar, lecture)					56	24.45
Pain in the social environment					229	100
No					57	24.89
I don't know					31	13.54
Yes, but I don't notice it much					91	39.74
Yes, and I engage with it					50	21.83
Acute pain status					229	100
Yes					59	25.76
No					170	74.24
Intensity acute pain (0 “weakest pain”–10 “most severe pain”)	3.64	1.77	0	8	59	25.76
Body areas acute pain						
Head					17	28.81
Stomach					14	23.73
Spine					26	44.07
Joint					17	28.81
Extremities					11	18.64
Others					11	18.64
Chronic pain status^1^					229	100
Yes					95	41.48
No					134	58.52
Intensity chronic pain (0 “weakest pain”–10 “most severe pain”)	4.56	1.89	0	9	95	41.58
Body areas chronic pain						
Head					40	42.11
Stomach					26	27.37
Spine					42	44.21
Joint					19	20.00
Extremities					12	12.63
Others					16	16.84
Frequency chronic pain	2.68	0.96	1	5	95	41.58
No pain last 4 weeks					1	1.05
Less than once a day					50	52.63
Once a day					23	24.21
Most of the day					17	17.89
Always					4	4.21
Pain knowledge variables						
Self-rated general pain knowledge (0 “low general pain knowledge”–100 “very high general pain knowledge”)	41.17	23.36	0	100	229	100
NPQ-d^2^ (0 “low neurophysiological knowledge”–1 “high neurophysiological knowledge”)	0.589	0.128	0.17	0.92	229	100

^1^Chronic (primary) pain was defined as pain at least once a month in the last 6 months or persistent pain.

^2^Neurophysiology of Pain Questionnaire (German version).

**Table 2:** Model fits of latent profile analysis for biopsychosocial pain concepts.

Profiles	LogLik	AIC	BIC	SABIC	Entropy	BLRT_val	BLRT_p	LMR LR	LMR_p
*Equal variances, covariances fixed to zero*									
1	−246.29	504.57	525.17	506.16	1.00	NA	NA	NA	NA
2	−183.22	386.43	420.77	389.08	0.68	126.14	**0.0099**	126.14	**0.001**
3	−149.66	327.32	375.39	331.02	**0.81**	67.12	**0.0099**	67.12	**0.001**
4	−139.26	314.52	376.33	319.28	0.74	20.80	**0.0198**	20.80	**0.012**
5	−127.40	**298.81**	**374.35**	**304.63**	**0.78**	23.71	**0.0198**	23.71	**0.004**
6	−125.54	303.08	392.36	310.00	0.77	3.73	0.6634	3.73	0.898
7	−123.85	307.70	410.71	315.63	0.71	3.39	0.7822	3.39	0.922
8	−121.28	310.57	427.32	319.56	0.72	5.13	0.5347	5.13	0.775
9	−125.04	326.08	456.56	336.12	0.71	−7.51	0.8119	−7.51	1.000

*Varying variances and covariances*									
1	−143.54	305.08	**335.99**	**307.46**	1.00	*NA*	*NA*	*NA*	*NA*
2	−133.20	304.41	369.65	309.43	0.44	20.67	0.3564	20.68	0.147
3	−125.87	309.73	409.31	317.40	0.52	14.67	0.6535	14.67	0.463
4	−112.05	**302.10**	436.01	312.41	0.63	27.64	0.2772	27.64	**0.026**
5	−113.41	324.83	493.08	337.78	0.77	−2.73	0.8812	−2.73	1.000
6	−93.89	305.78	508.37	321.38	0.76	39.05	0.1386	39.05	0.001
7	−92.53	323.07	560.00	341.31	0.75	2.71	0.7129	2.71	1.000
8	−87.04	332.07	603.34	352.96	0.77	11.00	0.7030	−0.00	1.000
9	**−67.59**	313.17	618.77	336.70	**0.78**	38.90	0.1485	38.90	0.001

*Note:* LogLik = Log-likelihood, SABIC = adjusted BIC, BLRT_val = parametric bootstrapped likelihood ratio test value, LMR LR = Vuong–Lo–Mendell–Rubin likelihood ratio test, LMR_p = LMRT probability. Bold values represent best model fits.

Abbreviations: AIC = Akaike information criterion, BIC = Bayes information criterion, BLRT_p = BLRT probability.

**Table 3:** Model estimated sizes, indicator means, and indicator standard deviations for the five latent classes.

Variable	Profile 1 (*strongly developed biopsychosocial pain concept*)	Profile 2 (*weakly developed biopsychosocial pain concept*)	Profile 3 (*psychosocially dominated pain concept*)	Profile 4 (*moderate biopsychosocial pain concept*)	Profile 5 (*weakly developed psychosocial pain concept*)
Profile size	*n* = 32 (13.97%)	*n* = 6 (2.62%)	*n* = 76 (33.19%)	*n* = 102 (44.54%)	*n* = 13 (5.68%)
Indicator variables					
Biological subscale (0–4)	*M* = 3.11 (SD = 0.23),	*M* = 2.27 (SD = 0.43),	*M* = 2.78 (SD = 0.24),	*M* = 2.63 (SD = 0.23),	*M* = 2.71 (SD = 0.29),
Psychological subscale (0–4)	*M* = 3.47 (SD = 0.20)	*M* = 1.94 (SD = 0.21)	*M* = 3.15 (SD = 0.18)	*M* = 2.78 (SD = 0.20)	*M* = 2.59 (SD = 0.22)
Social subscale (0–4)	*M* = 3.53 (SD = 0.16)	*M* = 2.19 (SD = 0.17)	*M* = 3.22 (SD = 0.19)	*M* = 2.84 (SD = 0.20)	*M* = 2.27 (SD = 0.14)
Demographic variables					
Age	*M* = 23.47 (SD = 5.97)	*M* = 22.17 (SD = 1.94)	*M* = 22.58 (SD = 2.98)	*M* = 22.32 (SD = 3.03)	*M* = 23.92 (SD = 4.01)
Sex					
Male	*n* = 7 (21.9%)	*n* = 2 (33.3%)	*n* = 21 (27.6%)	*n* = 21 (20.6%)	*n* = 5 (38.5%)
Female	*n* = 25 (78.1%)	*n* = 4 (66.7%)	*n* = 55 (72.4%)	*n* = 80 (78.4%)	*n* = 8 (61.5%)
Divers	*n* = 0	*n* = 0	*n* = 0	*n* = 1 (1.0%)	*n* = 0
Pain-related variables					
Previous pain education					
No	*n* = 15 (46.9%)	*n* = 4 (66.7%)	*n* = 50 (65.8%)	*n* = 65 (63.7%)	*n* = 9 (69.2%)
Yes (professional experience)	*n* = 9 (28.1%)	*n* = 0	*n* = 9 (11.8%)	*n* = 12 (11.8%)	*n* = 0
Yes (seminar, lecture)	*n* = 8 (25.0%)	*n* = 2 (33.3%)	*n* = 17 (22.4%)	*n* = 25 (24.5%)	*n* = 4 (30.8%)
Pain environment					
No	*n* = 6 (18.8%)	*n* = 1 (16.7%)	*n* = 18 (23.7%)	*n* = 31 (30.4%)	*n* = 1 (7.7%)
I don't know	*n* = 5 (15.6%)	*n* = 1 (16.7%)	*n* = 7 (9.2%)	*n* = 16 (15.7%)	*n* = 2 (15.4%)
Yes, but I don't notice it much.	*n* = 14 (43.8%)	*n* = 2 (33.3%)	*n* = 35 (46.1%)	*n* = 35 (34.3%)	*n* = 5 (38.5%)
Yes, and I engage with it.	*n* = 7 (21.9%)	*n* = 2 (33.3%)	*n* = 16 (21.1%)	*n* = 20 (19.6%)	*n* = 5 (38.5%)
Acute pain status (yes/no)	*n* = 14 “yes” (43.8%)	*n* = 2 “yes” (33.3%)	*n* = 16 “yes” (21.1%)	*n* = 25 “yes” (24.5%)	*n* = 2 “yes” (15.4%)
Intensity acute pain (1 “weakest pain”−10 “most severe pain”)	*M* = 3.42 (SD = 1.10)	*M* = 3.43 (SD = 0.33)	*M* = 1.76 (SD = 0.46)	*M* = 3.68 (SD = 0.81)	*M* = 3.26 (SD = 1.09)
Chronic pain status (yes/no)	*n* = 14 “yes” (43.8%)	*n* = 3 “yes” (50.0%)	*n* = 33 “yes” (43.4%)	*n* = 37 “yes” (36.3%)	*n* = 8 “yes” (61.5%)
Intensity chronic pain (1 “weakest pain”−10 “most severe pain”)	*M* = 1.56 (SD = 0.50)	*M* = 3.61 (SD = 1.42)	*M* = 4.75 (SD = 1.32)	*M* = 4.63 (SD = 1.06)	*M* = 4.21 (SD = 1.61)
Frequency chronic pain (1 “no pain in the last 4 weeks”–5 “always”)	*M* = 3.07 (SD = 0.92)	*M* = 2.67 (SD = 1.16)	*M* = 2.82 (SD = 0.98)	*M* = 2.54 (SD = 0.87)	*M* = 2.13 (SD = 1.13)
Other, pain-related knowledge matters					
Self-rated general pain knowledge (0 “low general pain knowledge”–100 “very high general pain knowledge”)	*M* = 53.50 (SD = 23.53)	*M* = 32.00 (SD = 17.72)	*M* = 40.24 (SD = 23.71)	*M* = 38.51 (SD = 21.89)	*M* = 41.38 (SD = 27.30)
Neurophysiological knowledge (0 “low neurophysiological knowledge−1 “high neurophysiological knowledge”)	*M* = 0.65 (SD = 0.11)	*M* = 0.597 (SD = 0.17)	*M* = 0.62 (SD = 0.12)	*M* = 0.58 (SD = 0.13)	*M* = 0.51 (SD = 0.14)

*Note: M* = mean value.

Abbreviation: SD = standard deviation.

**Table 4:** Multiple regression models using person characteristics to predict class membership probabilities.

	Predictor	Profile 1 (*strongly developed biopsychosocial pain concept*)				Profile 2 (*weakly developed biopsychosocial pain concept*)				Profile 3 (*psychosocially dominated pain concept*)				Profile 4 (*moderate biopsychosocial pain concept*)				Profile 5 (*weakly developed psychosocial pain concept*)			
		*B*	SE	*ß*	*p*	*B*	SE	*ß*	*p*	*B*	SE	*ß*	*p*	*B*	SE	*ß*	*p*	*B*	SE	*ß*	*p*
Research question 2a	Age	0.008	0.005	0.096	0.151	0.001	0.003	0.024	0.721	0.002	0.007	0.020	0.768	0.011	0.008	0.096	0.151	0.002	0.004	0.037	0.582
	Sex	0.042	0.044	0.064	0.341	0.014	0.023	0.042	0.531	0.024	0.055	0.029	0.662	0.037	0.062	0.040	0.553	0.040	0.033	0.083	0.216

Research question 2b	Previous pain education	0.012	0.035	0.036	0.726	0.032	0.019	0.173	0.099	0.003	0.046	0.008	0.943	0.016	0.050	0.032	0.757	0.039	0.032	0.126	0.230
	Pain environment	0.006	0.029	0.022	0.835	0.020	0.016	0.135	0.208	0.028	0.039	0.079	0.464	0.092	0.042	0.229	0.032	0.050	0.027	0.199	0.067
	Acute pain	0.020	0.063	0.036	0.748	0.006	0.035	0.021	0.853	0.155	0.083	0.215	0.064	0.142	0.091	0.176	0.120	0.014	0.057	0.027	0.812
	Intensity acute pain^1^	0.027	0.018	0.167	0.129	0.002	0.014	0.013	0.914	0.050	0.034	0.173	0.146	0.012	0.037	0.038	0.742	0.018	0.024	0.087	0.463
	Frequencies chronic pain	0.088	0.037	0.280	0.019	0.000	0.020	0.001	0.992	0.039	0.048	0.098	0.418	0.105	0.053	0.233	0.051	0.023	0.033	0.081	0.503
	Intensity chronic pain	0.027	0.018	0.167	0.129	0.014	0.010	0.162	0.152	0.001	0.023	0.004	0.969	0.055	0.025	0.238	0.033	0.013	0.016	0.092	0.412

Research question 2c	NPQ-d	0.384	0.150	0.208	**0.011**	0.029	0.081	0.024	0.722	0.359	0.196	0.125	0.068	0.430	0.219	0.132	0.051	0.343	0.114	0.202	**0.003**
	Self-rated general pain knowledge	0.002	0.001	0.175	**0.008**	0.000	0.000	0.056	0.417	0.001	0.001	0.033	0.624	0.001	0.001	0.082	0.228	0.000	0.001	0.016	0.809

*Note:* Alpha-levels were adjusted to 0.016 to reduce the risk of alpha-error accumulation. Significant results are printed in bold.

^1^The variable “chronic pain” is not depicted due to a zero correlation with the criteria.

**Table 5:** Structure of the BiPS matrix (translated version without psychometric testing).

BiPS matrix item	Content dimension according to CSM	Biopsychosocial domain
1. Chronic pain has a warning function.	Illness identity	Biological
2. Acute pain has a protective function for the body.	Illness identity	Biological
3. Special nerves in the spinal cord send danger signals to the brain.	Illness identity	Biological
4. If you have been in pain for several months, the brain becomes more sensitive to warning signals.	Illness identity	Biological
5. The brain processes many details before deciding when to feel pain.	Illness identity	Psychological
6. Thoughts can influence the intensity of pain.	Illness identity	Psychological
7. The intensity of pain is independent of what you know about pain.	Illness identity	Psychological
8. The intensity of pain changes when you argue with your partner, family, or a good friend.	Illness identity	Social
9. The intensity of pain remains the same regardless of where and with whom you are.	Illness identity	Social
10. The intensity of pain differs between people from different cultures.	Illness identity	Social
11. More severe injuries always cause more severe pain.	Cause	Biological
12. Pain only occurs when you are injured or at risk of injury.	Cause	Biological
13. If for fear of pain you don't move at all or don't move much, muscles are broken down. This leads to more pain.	Cause	Biological
14. You feel more pain when you are worried about your pain.	Cause	Psychological
15. Suppressed anger or sadness can cause pain to become more severe.	Cause	Psychological
16. The way you think about your pain does not change the pain.	Cause	Psychological
17. Education about pain can make the pain less severe.	Cause	Social
18. Constant attention from a partner, friends, or family to persistent pain can make the pain worse.	Cause	Social
19. If parents react anxiously when a child is injured, the pain may become worse.	Cause	Social
20. Too much pain medication can cause the pain to persist.	Consequences	Biological
21. In the case of acute pain, operations to treat the cause of the pain can lead to pain relief.	Consequences	Biological
22. If you have chronic pain, you may feel helpless or hopeless.	Consequences	Psychological
23. Chronic pain often leads to impairments in the daily lives of those affected.	Consequences	Psychological
24. You can do things together with friends, your partner, or family despite chronic pain.	Consequences	Social
25. Chronic pain can cause you to withdraw from friends, family, or your partner more often.	Consequences	Social
26. Pain can mean that you can no longer go to work.	Consequences	Social
27. Acute pain usually has a clear trigger. After healing, the pain decreases.	Timeline	Biological
28. When an injury has healed properly, there is no more pain.	Timeline	Biological
29. Being happy can reduce the pain.	Timeline	Psychological
30. If you have chronic pain, you always feel pain.	Timeline	Psychological
31. The pain remains the same throughout the day, regardless of who you spend the day with.	Timeline	Social
32. You always have to take medication to treat acute pain.	Control	Biological
33. It is not always necessary to take medication to deal with chronic pain.	Control	Biological
34. Sport and exercise help to reduce chronic pain.	Control	Biological
35. Distraction can reduce pain.	Control	Psychological
36. Pleasant activities (e.g., listening to music) do not reduce pain.	Control	Psychological
37. It is easier to deal with pain if you relax.	Control	Psychological
38. With chronic pain, it is good to meet up with friends, your partner, or family and continue to pursue your hobbies.	Control	Social
39. Parents have no influence on children's chronic pain.	Control	Social
40. Treatment teams (e.g., consisting of psychotherapists, doctors, physiotherapists, nurses) can help to reduce the intensity of pain.	Control	Social

## Data Availability

The authors are not able to make the data or scripts of the project available, given that consent for data sharing was not obtained at the beginning of the study in 2021.

## Appendix A: Identifying Latent Profiles of Healthy Adults' Biopsychosocial Pain Concepts

*Structure of the BiPS Matrix (Translated Version Without Psychometric Testing)*

*Sociodemographic and Pain-Related Questionnaire*

1. How old are you?
  • Open answer format
  • Filter question: The survey is automatically terminated for numbers under 18 years of age
2. Please indicate your sex.
  • Selection female/male/diverse
3. Are you currently studying?
  • yes—no
  • Filter question: if “no,” the survey will end automatically
4. Which stage of your studies are you currently in?
  • Bachelor, semester ______
  • Master, semester _______
5. What is your field of study?
  • Psychology, Law, Business Administration, German Studies, Educational Science, another subject: ______________________ (open answer format)
6. What is your current living situation?
  • Selection: alone, with family, with partner, shared flat, other
7. How did you become aware of the study? Several answer options:
  • Trier University e-mail distribution list
  • SONA system of Trier University
  • Information from courses at Trier University
  • Social media (e.g., Facebook, Instagram)
  • Private contacts
  • Other: (field for input)
Self-rated general pain knowledge:
8. On a scale from 0 (no general pain knowledge) to 100 (very high general pain knowledge), how high would you rate your general pain knowledge?
  • Scale 0–100 (slider)
9. Do you have professional experience in the field of pain or have you attended a lecture or seminar on the subject of pain?
  • no; yes, professional experience; yes, attended a lecture/seminar
Pain-related variables:
10. Does anyone in your family, circle of friends, or immediate environment suffer from recurring or chronic pain (i.e., pain at least once a month in the last 6 months or persistent pain)?
  • no; I don't know; yes, but I don't notice much of it; yes and I am dealing with the topic
11. Do you currently suffer from pain?
  • Yes - no
  • Filter question, if “no,” questions 12 and 13 are hidden
12. How strong is your current pain on a scale from 1 (weakest pain) to 10 (most severe pain)?
  • Scale 1–10
13. Where is your current pain localized (multiple answers possible)?
  • head, abdomen, back, joints, extremities
  • other___________________
14. Do you suffer from recurring or chronic pain, i.e., have you had pain at least once a month in the last 6 months or persistent pain?
  • Yes - no
  • Filter question, if “no,” questions 15–19 are hidden
    The following questions relate to your chronic pain.”
15. On a scale from 1 (weakest pain) to 10 (most severe pain), how severe was your pain most of the time in the last four weeks?
  • Scale 1–10
16. How often did your (main) pain generally occur (on average) in the last 4 weeks?
If pain occurs in several places, the main pain is the pain that significantly affects you.”
  • no pain in the last 4 weeks; pain less than once a day; pain maximum once a day; pain is always present except for rare pain-free moments and pain-free sleep; pain is constantly present
17. Where have you suffered from pain in the last 4 weeks?
  • Multiple answers possible
  • Head, abdomen, back, joints, extremities
  • Other _______________
18. Has your chronic pain prevented you from studying or working in the last 4 weeks?
  • Yes - no
19. Have you taken medication for your pain in the last 4 weeks?
  • No; once; once a week; several days a week; daily
20. How often have you sought treatment for pain-related reasons in the last year?
  • Enter number
  • If yes, what type of treatment ________________ (large free field for potential multiple answers)
